# Fine-Grained Assignment of Unknown Marine eDNA Sequences Using Neural Networks

**DOI:** 10.3390/biology15030285

**Published:** 2026-02-05

**Authors:** Sébastien Villon, Morgan Mangeas, Véronique Berteaux-Lecellier, Laurent Vigliola, Gaël Lecellier

**Affiliations:** 1 ENTROPIE, CNRS, Institute of Research for Development (IRD), University of New Caledonia, University of Reunion, IFREMER, Promenade Roger-Laroque, 98848 Noumea Cedex, New Caledonia, France; morgan.mangeas@ird.fr (M.M.); veronique.berteaux-lecellier@cnrs.fr (V.B.-L.); laurent.vigliola@ird.fr (L.V.);; 2 CECI, Centre Européen de Recherche et de Formation Avancée en Calcul Scientifique, CNRS, Institute of Research for Development (IRD), Université de Toulouse, 31100 Toulouse, France; 3 EMR9001 SantEco, CNRS, Institute of Research for Development (IRD), University of New Caledonia, University of Reunion, Promenade Roger-Laroque, 98848 Noumea Cedex, New Caledonia, France; 4 Institut de Sciences Exactes et Appliquées (ISEA) EA7484, 145, Avenue James Cook—BP R4, 98851 Noumea Cedex, New Caledonia, France

**Keywords:** biodiversity monitoring, environmental DNA, bioinformatics, deep learning algorithms, convolutional neural networks CNN, fish diversity, ecological survey

## Abstract

Environmental DNA (eDNA) metabarcoding is an innovative tool that is transforming ecological research. It offers a simple and effective method for simultaneously detecting numerous species across a wide range of environments. The method relies on assigning DNA sequences sampled from the environment to taxa, which is straightforward for species that have already been sequenced and are represented in reference databases. However, existing bioinformatics tools often fail to deliver accurate, fine-grained assignments when target species are absent from these databases. This limitation arises from handcrafted classification thresholds that do not account for nucleotide positional information. Here, we propose a deep neural architecture specifically designed to exploit both nucleotide identity and positional patterns in short TELEO sequences. Using an in-silico validation framework based on NCBI genbank sequences, we compare our approach with several state-of-the-art bioinformatics tools (Obitools, Kraken2, Lolo), as well as alternative sequence embedding methods, under controlled conditions. Our approach yields significantly higher classification accuracy at the genus and family levels, achieving average accuracies of 94.7% at the genus level and 86.5% at the family level, substantially outperforming the tested reference-based pipelines. The method remains robust with limited training data and shows improved performance when nucleotide positional information is preserved through sequence alignment. These results demonstrate the potential of AI-powered eDNA metabarcoding to complement existing taxonomic assignment tools, particularly in contexts where reference databases are incomplete or species-level resolution is not achievable, thereby supporting biodiversity monitoring and ecosystem management.

## Simple Summary

Environmental DNA (eDNA) metabarcoding is increasingly used to monitor biodiversity by detecting traces of DNA left by organisms in the environment. While this approach allows the simultaneous detection of many species, its effectiveness is often limited by incomplete reference databases, especially in marine ecosystems. As a result, species-level identification is frequently unreliable or impossible, leading to large proportions of unassigned sequences. In this study, we propose a deep learning-based approach designed to improve taxonomic assignment at higher levels, such as genus and family, even when species are absent from reference databases. By learning patterns directly from DNA sequences, our method provides more accurate and consistent assignments than commonly used bioinformatic tools under constrained conditions. Although species-level identification remains essential when feasible, reliable genus- and family-level information already supports many ecological applications, including functional analyses, community comparisons, and long-term monitoring. Our results highlight the potential of artificial intelligence to complement existing eDNA tools and enhance biodiversity assessments in data-limited contexts.

## 1. Introduction

Environmental DNA (eDNA) has emerged as a valuable tool for studying fish communities. It contributes to our understanding of marine biodiversity and the conservation of marine ecosystems [1]. eDNA refers to the genetic material shed by organisms into their surrounding environment, such as water or sediment. By extracting and analyzing this genetic material, researchers can gain insights into the presence, abundance, and diversity of fish species, without the need for direct observation or capture [2]. Thus, eDNA analysis offers a non-invasive and highly efficient alternative to traditional biodiversity survey methods. Compared to such methods (e.g., visual surveys) or experimental fishing, eDNA allows researchers to survey larger areas and detect rare or elusive species. This is particularly relevant in marine environments such as coral reefs and mesopelagic zones, where biodiversity is high but direct observation is logistically challenging and often incomplete. In such areas, eDNA provides a valuable non-invasive alternative for biodiversity surveys. The methods to analyze eDNA samples consist of DNA extraction, polymerase chain reaction (PCR) amplification of target genes or regions, DNA sequencing or metabarcoding, and bioinformatics to process the raw data and identify the fish species present. However, eDNA analysis is not without limitations. The sensitivity of detection can vary depending on several factors, such as water flow or the distance from the organism’s source [3,4,5]. Another challenge for eDNA analysis is the degradation rate of DNA. DNA persists in aquatic environments for a limited time (typically hours to days), with decay rates influenced by temperature, microbial activity, and other environmental factors. eDNA analysis may also encounter challenges in species identification when dealing with closely related or cryptic species that share similar genetic markers [6]. More generally, taxonomic assignment is limited by the length of the amplicons, the accuracy of the sequencing technology (Illumina, Nanopore, Minion), and the heavy reliance on the reference database used by the bioinformatic pipeline. Furthermore, thresholds for taxonomic assignment are often set arbitrarily and may prevent accurate taxa identification. For example, depending on the selected marker gene, the geographical region, and the reference database, the proportion of unassigned sequences can reach up to 90%, of which 40% to 90% belong to microorganisms [7,8,9]. These limitations are particularly problematic in marine ecosystems, where many species remain underrepresented in public reference databases. In such cases, genus- or family-level assignment often becomes the most practical solution for ecological inference. To overcome the incompleteness of DNA reference databases and enhance the identification of unknown operational taxonomic units (OTUs) or amplicon sequence variants (ASVs), deep learning, a branch of artificial intelligence, holds promise for addressing some of these challenges [10,11,12]. Indeed, deep learning algorithms can learn from existing data to recognize patterns and make accurate predictions on new data. Applied to existing DNA databases, this approach has the potential to improve identification accuracy and overcome the limitations associated with traditional eDNA metabarcoding methods, thereby further enhancing our confidence in eDNA-based estimates of fish distribution and abundance in aquatic ecosystems. In many ecological contexts, particularly in biodiversity monitoring or conservation programs, genus- or family-level resolution is sufficient to assess community composition or detect shifts over time. Improving the accuracy of taxonomic assignment at these ranks can therefore have practical implications for ecological decision-making. While machine learning and probabilistic models have previously been used in taxonomic assignment, most studies have focused on species-level predictions. Furthermore, few have addressed the case of very short amplicon sequences in the presence of sparse and incomplete references—conditions that are common in marine eDNA applications. This study builds upon existing methods by exploring a convolutional neural network (CNN) architecture trained on short TELEO sequences. The objective is to improve assignment accuracy at the genus and family levels, even when the target species is absent from the reference dataset. By learning position-aware patterns in the sequences, this approach is designed to enhance taxonomic inference in constrained ecological settings.

In this paper, we build a deep-learning network specifically designed to process eDNA samples extracted with the widely-used genetic primer TELEO [13]. Then, we compare the outcomes of our model with those from three widely-used bioinformatic pipelines, Kraken2 [8,14], Obitools [15] and Lolo [16]. We compared all methods in an in silico validation framework. Here, “in silico” denotes analyses performed exclusively on computational datasets derived from reference DNA sequence databases (NCBI, National Center for Biotechnology Information), without using new environmental or laboratory samples.

The comparisons specifically focused on identifying eDNA sequences at the genus and family levels that are missing from training or reference DNA databases, which contain species-level data. While this study focuses on convolutional neural networks, recent advances in transformer-based sequence models offer promising perspectives for eDNA sequence analysis and could be explored in future work.

## 2. Materials and Methods

### 2.1. Teleo Fish Dataset

For this experiment, we constructed a subset of fish DNA sequences from the NCBI (National Center for Biotechnology Information) genbank database. This subset was obtained with the Basic Local Alignment Search Tool (BLAST) (https://blast.ncbi.nlm.nih.gov/Blast.cgi?PROGRAM=blastx&PAGE_TYPE=BlastSearch&LINK_LOC=blasthome accessed on 28 January 2026) [17] with an evaluation parameter of 10^−5^, to find the region of local similarity between sequences and the “teleo” region of 12S, used in many eDNA analyses [18,19,20,21,22]. After removing duplicates, this subset contained 4739 distinct species-sequences pairs belonging to the fish classes *Actinopteri* and *Chondrichthyes*. Subsequently, from this subset, two distinct datasets were created to assess our method’s performance. In both datasets, we exclusively considered genera and families that contained at least 20 sequences. This threshold was chosen empirically as a compromise between having a sufficient number of sequences per class to allow stable CNN training and maintaining a broad coverage of genera and families in the dataset. The family dataset (raw-family) consisted of 50 families, encompassing 21 to 425 sequences per family (Supplementary Table S3). The genus dataset (raw-genus) comprised 17 genera, with each genus containing between 23 and 53 sequences (Supplementary Table S2).

### 2.2. Filled and Aligned Datasets

Both the genus and family datasets comprised DNA sequences ranging in length from 23 to 83 nucleotides, with each position able to adopt one of four letters: A, C, T, or G (Table 1 (A)). Two methods were tested for the analysis of sequences into our convolutional neural network (CNN) architecture.

The first method consisted of obtaining a uniform length of 83 nucleotides for each sequence, adding a fifth letter, “n,” to the end of shorter sequences (Table 1 (B)).

The second method consisted of performing a multiple sequence alignment by Clustal Omega (BioPython 1.76) [23] with standard parameters on the sequences of the subset, to obtain a uniform length, but with insertions of “n” inside the sequences of the aligned datasets (Table 1 (C)).

We performed both methods on our two raw datasets (family and genus) to obtain four datasets (filled_genus, aligned_genus, filled_family, and aligned_family) amenable to deep-learning modeling.

### 2.3. CNN Architecture

Prior to being fed into our CNN architecture, each DNA sequence underwent transformation into five binary vectors (Table 2). These vectors represent the presence or absence of each nucleotide at each position.

**Figure 1:**
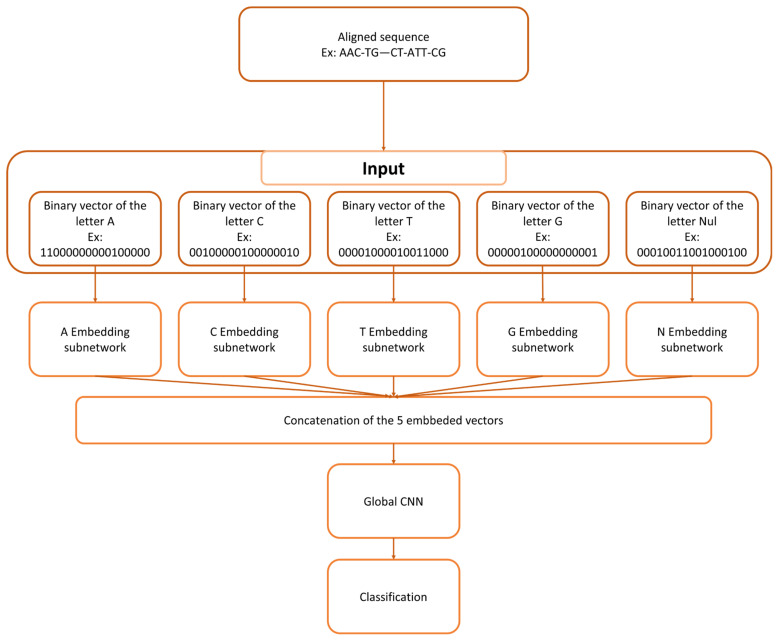
Diagram of our architecture. (1) The sequence is transformed into binary 5 vectors, each representing the presence/absence one of the 5 possibilities: A, C, T, G, -; (2) Each vector is fed to a CNN; (3) Each CNN output is concatenated, therefore creating one vector (4) this new vector is fed to a CNN (5) the output is compared to the expected result, and the error is retro-propagated at once though the common and the specific parts of the network.

The CNN architecture was defined based on preliminary hyperparameter exploration conducted prior to the main experiments. Kernel sizes ranging from 3 to 12 and numbers of convolutional filters ranging from 32 to 512 were tested in exploratory runs, as well as the number of convolutional, pooling, and dropout layers. Smaller kernels consistently provided stable performance on short TELEO sequences, while increased filter depth improved representational capacity. Our final configuration consisted of five individual heads, with each head processing a specific binary word (Figure 1). The architecture was kept light, with limited data reduction through pooling and dedicated heads in order to be efficient in processing short amplicons. Each head comprised five convolutional layers, a pooling layer, and a dropout layer. The outputs from the five heads were merged into the main body of the architecture, which consisted of one convolutional layer, followed by two dense layers. Each convolutional layer was composed of 256 kernels of size 3. The final layer was a softmax classification layer. The training phase lasted 30 epochs, where each epoch involved processing the entire training database. Each epoch was divided into batches of 32 sequences. On average, the training of a model lasted approximately 63 s, utilizing two Nvidia Quadro GPUs.

### 2.4. K-Fold Methodology and Metrics Computing

For our comparative evaluation, we conducted a 100-fold experiment, where each family and genus dataset was randomly split into 80% for training, and 20% for testing in each fold with its own distinct sequence. These 200 training and testing dataset pairs were subsequently processed for taxonomic assignment.

For a given genus or family, accuracy was defined as the number of sequences originating from that taxon that were correctly assigned to the same taxonomic rank, divided by the total number of test sequences belonging to that taxon. Accuracy values were first computed independently for each genus or family for each replicate and then averaged across replicates. This per-taxon (macro-averaged) accuracy prevents large taxa from dominating the results and allows a fair comparison across methods under class-imbalanced conditions.

### 2.5. BioInformatics Tools

Taxonomic assignments of the sequences from the testing sets were carried out using three widely used methods to be compared with the CNN architecture:

(1) The Obitools method consists of the Lower Common Ancestor (LCA) algorithm ecotag, implemented in the Obitools toolkit [15] with the training set as the reference database. In this experiment, we used the implementation of Obitools2.
(2) The Lolo method [16] consists of the previous results from the Lower Common Ancestor (LCA) algorithm ecotag implemented in the Obitools, assigning to the upper taxonomic level common between all possible matches, and filtering according to the taxonomic level. Assignment was accepted at the species level when the percentage of similarity with the reference sequence was 100%, at the genus level between 90 and 99% similarity, and at the family level at a similarity between 85% and 90%. If these criteria were not met, the sequence was left unassigned. The threshold of 85% similarity for family assignment was chosen to include a maximum of correct family assignments while minimizing the risk of adding wrong family assignments in the family detections.
(3) The Kraken2 method uses a k-mer-based approach [14]. It breaks DNA sequences into short, fixed-length k-mers (typically 31-mers) and compares these k-mers to the sequences in the reference database. Each k-mer is associated with a taxonomic label based on the database. This processing is then linked to an LCA Algorithm to determine the most specific taxonomic label that is common to the majority of k-mers in a sequence. This reduces false positives by considering the taxonomic label that represents the most specific common ancestor of the k-mers.

### 2.6. Statistical Analysis

First, we used paired *t*-tests to compare the effect of sequence preparation (filled vs. aligned) on classification accuracy. The comparison was performed within each taxonomic level (genus or family), using paired values for each taxon, to evaluate whether sequence alignment improved classification performance compared to simple length normalization. To assess whether classification accuracy differed significantly across the different methods tested, we performed two one-way ANOVAs, one for genus and one for family. The objective was to test whether the method used (CNN, Kraken2, Obitools, or Lolo) had a significant effect on assignment accuracy, averaged over 100 replicates for each taxon. The mean accuracy per genus or family was used as the response variable. For the ANOVA analyses, normality of residuals was assessed using Shapiro–Wilk tests and Q–Q plots. While the Shapiro–Wilk test indicated deviations from strict normality, visual inspection showed that residuals were approximately symmetric with departures mainly in the tails. Homoscedasticity was evaluated using Levene’s test and indicated unequal variances across groups. Given the large sample size, balanced design, and the magnitude of observed differences between methods, the ANOVA was considered robust to these deviations. A post hoc Tukey HSD test was used to identify pairwise differences between methods.

## 3. Results

### 3.1. Comparison Between Aligned and Filled Data

Our proposed method showed a significantly better accuracy on family assignment with aligned data, with an average accuracy of 86.51 ± 1.74% versus 82.76 ± 1.83% (paired *t*-test < 0.001). The genus assignment is slightly better with aligned data (Table 3), albeit not significantly (paired *t*-test > 0.05).

For further comparison between our proposal and other bioinformatics methods, we will only consider results for aligned data.

### 3.2. Sensitivity Analysis to Class Frequency

CNNs tend to work better with a large number of examples per class (in our case, per genus and family). However, it is unlikely to have hundreds of examples per class, as most genera and families do not contain hundreds of species. To assess the sensitivity of CNN to the amount of data available during training, we tried our architecture with 5, 10, 15, and 20 examples by class to assess how many examples were needed to make the use of CNN relevant. The thresholds impacted the number of genera/families with 50 families/17 genera above the threshold of 20, 62 families/28 genera above the threshold of 15, 91 families/65 genera above the threshold of 10, and 160 families/236 genera above the threshold of 5.

Our CNN proposal showed better accuracy than the compared BioInformatic tools methods with either 5, 10, 15, or 20 sequences per class. Since the highest accuracy was observed with 20 sequences per class (Table 4), this number was used in further experimentation.

### 3.3. Accuracy Comparisons Between CNN Proposal and Traditional Bioinformatic Methods

One-way ANOVAs revealed highly significant differences among models (*p*-value < 0.001) for both the family and the genus analyses. Post-hoc Tukey tests showed that our proposed method had significantly higher accuracy than Kraken2, Lolo, and Obitools (*p*-value > 0.05 for all tests).

**Figure 2:**
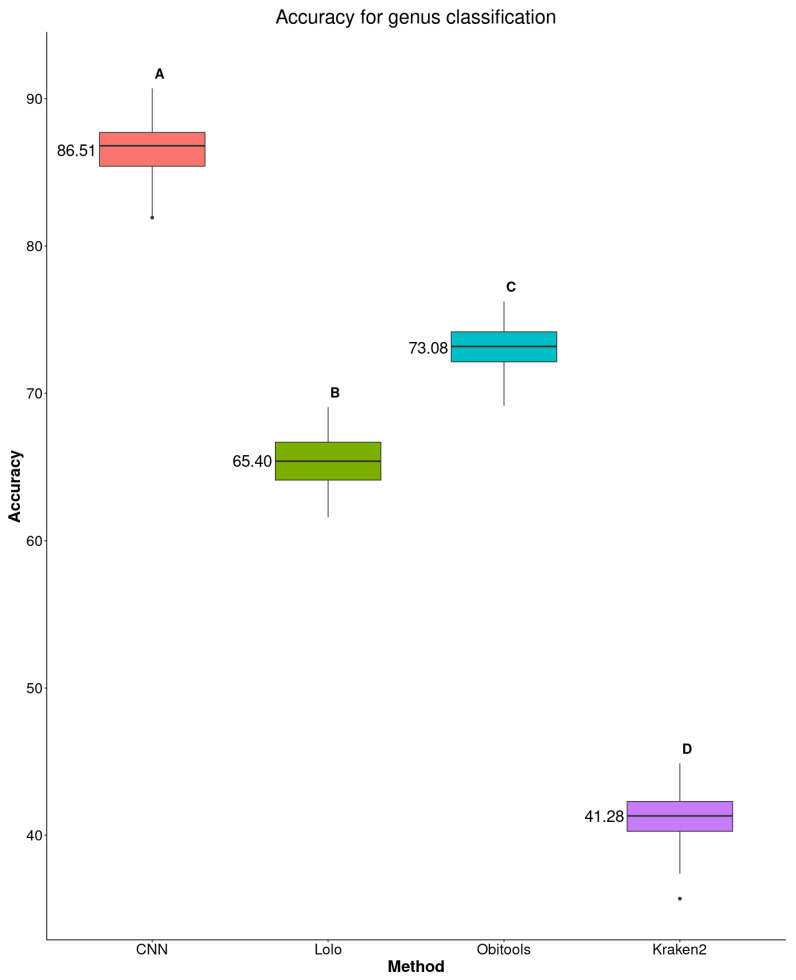
Comparison of sequence assignment accuracy at the family level, according to our proposal, Kraken2, Lolo, and Obitools. Different letters indicate significant differences at 5% in post-hoc Tukey tests. The average value of accuracy is mentioned on the left of each box.

Under our experimental conditions, the CNN approach achieved an average accuracy of 86.51% at the family level, compared to 73.08% for Obitools, 65.40% for Lolo, and 41.28% for Kraken2 (Figure 2 and Supplementary Table S1). These results should be interpreted in light of the scope of the dataset and the simplified taxonomic coverage.

**Figure 3:**
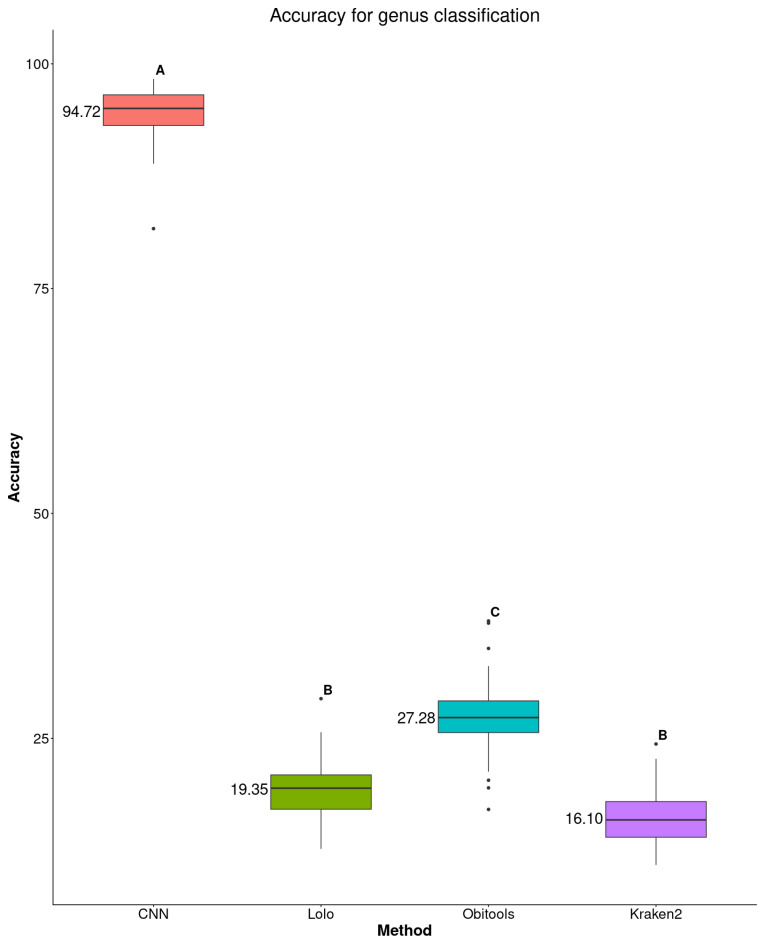
Comparison of sequence assignment accuracy at the genus level, according to our proposal, Kraken2. Lolo, and Obitools. Different letters indicate significant differences at 5% in post-hoc Tukey tests. The average value of accuracy is mentioned on the left of each box.

We also observed a dramatic improvement in accuracy for genus classification, with our method achieving an accuracy of 94.72%, while Obitools achieved only 27.28%, Lolo 19.35%, and Kraken2 16.10% (Figure 3 and Supplementary Table S1).

### 3.4. Accuracy Comparisons by Family and Genus

**Figure 4:**
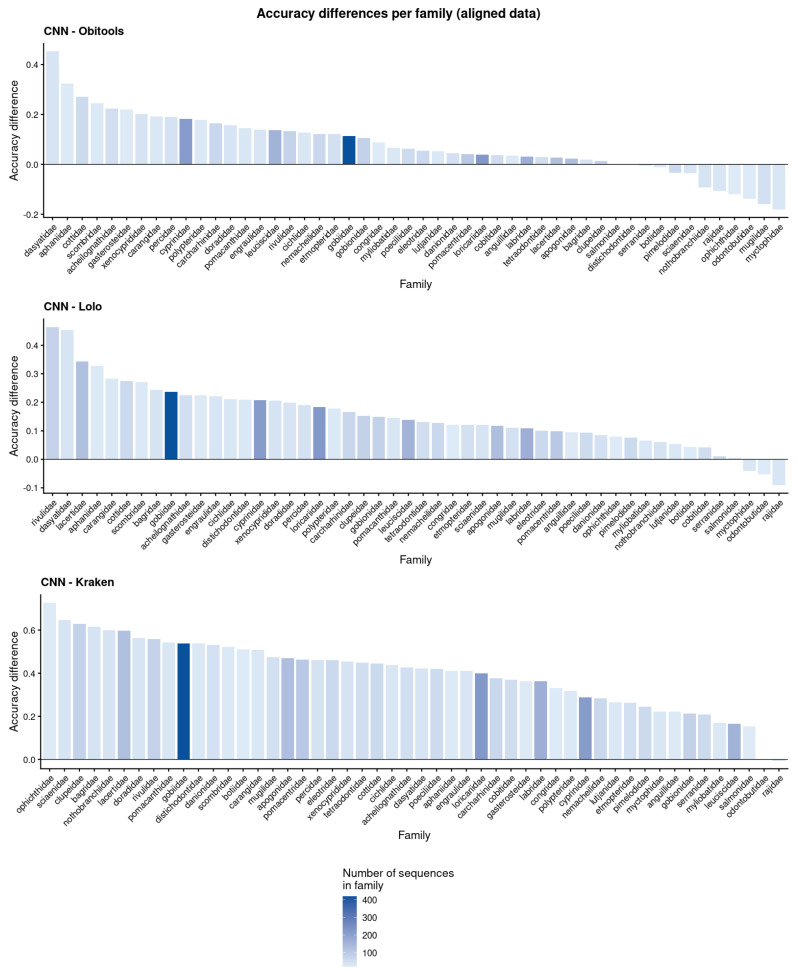
Accuracy difference between our proposal, Obitools, Lolo, and Kraken2 for families. Positive values show a better accuracy on the CNN, while negative values show better accuracy on other methods. The color gradient shows the number of sequences (testing + training) of each family. For a better visualization of families names, refer to Supplementary Figures S1–S3.

**Figure 5:**
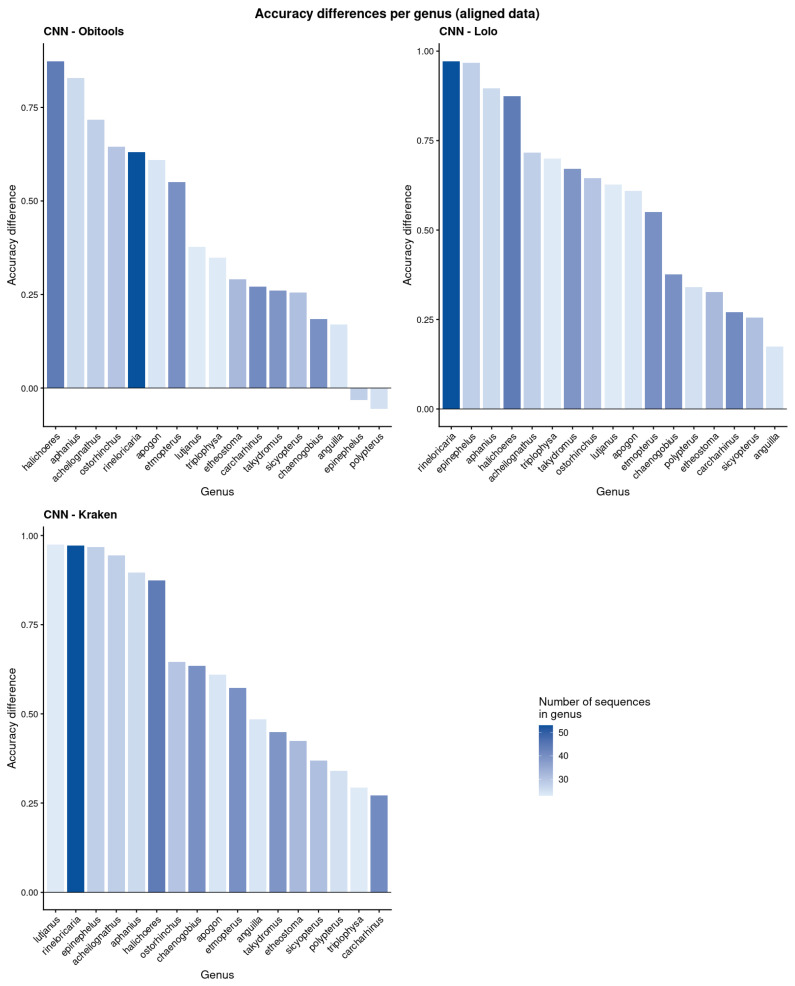
Accuracy difference between our proposal, Obitools, Lolo, and Kraken2 for genera. Positive values show better accuracy on the CNN, while negative values show better accuracy on other methods. The color gradient shows the number of sequences (testing + training) of each genus. For a better visualization of genus names, refer to Supplementary Figures S4–S6.

Comparing the results by taxa, the CNN outperformed Lolo’s accuracy on 47 out of the 50 families, and the positive differences reached 46% on *Rivulidae*, whereas the negative difference only reached 9% on *Rajidae*. The CNN also surpassed Obitools’ accuracy on 39 families, with a positive difference reaching 44% on *Dasyatidae*, while the top negative difference was 18% on *Myctophidae*. Finally, the CNN accuracy was greater than Kraken2 for 48 families, reaching an improvement of 73% at best with Ophichtidae, while Kraken2 reached a top accuracy difference with 0.6% on Rajidae (Figure 4). On genera classification we observed that the CNN accuracy was greater for all genera compared to Lolo and Kraken2 (Figure 5), while it was better than Obitools’ for 15 out of 17 genera (two genera reached a better accuracy with Obitools, *Epinephelus*, 3.2% and *Polypterus*, 5.6%).

## 4. Discussion

In this paper, we assessed the benefits of using CNN approaches to assign unknown sequences from eDNA samples to genus and family taxonomic ranks. These results are based on an in-silico validation framework using curated reference sequences, in which taxonomic absence was enforced computationally. No mock communities or environmental samples were processed, and empirical validation has yet to be conducted. From a taxonomic and ecological perspective, species-level identification is often considered the ideal outcome for biodiversity monitoring and management, particularly when precise species inventories or regulatory frameworks are required. However, such resolution critically depends on the completeness and quality of reference databases, a condition that is rarely met in many ecosystems, especially in marine environments and for understudied taxa. In these contexts, forcing species-level assignments may lead to overconfident or erroneous results. In contrast, genus- or family-level assignments represent a pragmatic and ecologically meaningful alternative when species-level resolution is unreliable. Many ecological applications, including functional ecology, trophic structure analyses, biogeographic assessments, and long-term monitoring of community composition, rely on higher taxonomic ranks that capture shared functional traits and evolutionary relationships. In such cases, obtaining accurate assignments at the genus or family level is preferable to uncertain species-level calls or unassigned sequences. The approach presented here is therefore not intended to replace species-level identification when it is feasible, but rather to complement existing methods by providing reliable higher-rank assignments in situations where reference databases are incomplete or species-level resolution cannot be achieved with confidence.

Our results indicate that the CNN approach performs well compared to three widely used taxonomic assignment tools under controlled experimental conditions. The model achieved good accuracy even when trained on relatively small reference subsets. The generalization of the model beyond the TELEO marker and the selected taxonomic groups remains to be evaluated. Performance may vary with marker region, sequencing technology, and environmental conditions.

Our experiments also explored the impact of the taxonomic rank on classification accuracy, and observed that accuracy improvement at genus rank was significantly higher than at family rank. At least two reasons may explain this difference: (1) the family database was composed of more classes, and hence exposed to a higher possibility of misclassification, and (2) the family dataset imbalances were greater, with sample sizes ranging from 425 to 21 between classes, whereas sample sizes ranged only from 53 to 23 for genera. The imbalance of datasets is a known issue for CNN classification models, but we decided that exploring data augmentation and focal loss was out of the scope of our demonstration. These results suggest that CNN-based classification may complement traditional taxonomic assignment tools, particularly in scenarios where reference completeness is limited or species-level assignment is not feasible. We also observed that this accuracy was reached with a very small training dataset, with a median of 37 sequences per genus or family.

As the incompleteness of eDNA reference databases is one of the biggest challenges for sequence classification, this method could allow an accurate classification of an unknown sequence to an upper taxonomic rank.

In our paper, we showed that the CNN model had better accuracy compared to Bioinformatic tools, even with only 5 sequences per class. We also showed that, on average, the increase in the number of sequences per genus or family implied an improved accuracy, but at the cost of reducing the number of genera and families in the database. Hence, such a choice between coverage and accuracy is up to the user, depending on the case study. For genus and species with fewer than five sequences, one-shot or few-shot methods [24,25] could be an alternative to classic CNNs training methods. It is important to note that existing bioinformatics tools are particularly efficient when the target sequences are identical or nearly identical to sequences present in the reference database, or when the number of closely related species within the same genus or family is low. In contrast, the approach presented here may offer improved performance in situations where the reference coverage is limited or incomplete, as it relies on learning sequence-level and positional patterns rather than strict identity matching. These methods can therefore be considered complementary, and their combined use may improve assignment reliability across a wider range of ecological contexts. Improved assignment accuracy at the genus and family levels may have important implications for downstream ecological analyses based on eDNA data. Many commonly used ecological metrics, such as taxonomic richness, community composition, and spatial or temporal turnover, rely on consistent assignment at intermediate taxonomic ranks when species-level resolution is not achievable. Increased accuracy at these levels may therefore reduce uncertainty in comparative analyses and improve the robustness of biodiversity indicators derived from eDNA surveys. The potential benefits of CNN-based assignment are expected to be most pronounced in ecological contexts where reference databases are sparse or uneven. These include understudied geographic regions and taxonomic groups that remain poorly represented in public databases. In such cases, reference-based methods may fail to provide reliable assignments beyond high taxonomic ranks, whereas learning-based approaches that exploit sequence-level and positional patterns may retain informative power at the genus or family level. On the other hand, when reference databases are complete and include closely related species, traditional bioinformatics tools based on sequence similarity or exact matching are expected to perform optimally, particularly at the species level. In such situations, the relative advantage of CNN-based approaches may be reduced.

On an important note, our results demonstrated that the CNN model achieved improved classification accuracy when working with aligned data. We also know that CNNs are efficient in detecting important positions in embedded information [26,27] This finding led us to the conclusion that the network not only considered nucleotide values but also weighted the position of the nucleotides within the sequence. As a result, the network enhanced the importance of sub-sequences shared by ancestors of two species within the same genus or family. If those extrapolations are right, the results of this study suggest that nucleotide positioning is an important factor in sequence assignment. These findings could have implications for the classification of bioinformatics tools, as the addition of positional penalties to specific and crucial sub-sequences may lead to further enhancements. Further research is underway to support these hypotheses and enhance sequence assignment.

eDNA is an ongoing revolution for marine ecology, but methods of assignment, as well as the scarcity of databases, are major bottlenecks to fully take advantage of this technology. While sequencing data are increasingly available, many taxa are still underrepresented or absent in public repositories, especially in marine environments. This limits the accuracy of taxonomic assignment, particularly for short amplicons and less-studied lineages.

Our work showed a lot of promise in the potential of deep learning to build new tools, especially to classify unknown sequences of DNA present in the water.

## 5. Conclusions

In this study, we investigated the potential of convolutional neural networks to improve the taxonomic assignment of eDNA sequences at the genus and family levels under conditions of incomplete reference databases. Using an in-silico validation framework based on curated reference sequences, in which taxonomic absence was enforced computationally, we showed that a position-aware CNN architecture can achieve higher assignment accuracy than commonly used bioinformatic tools when species-level identification is not achievable. From a taxonomic perspective, species-level identification remains the ideal objective when reference databases are sufficiently complete and well curated. However, such conditions are rarely met in many ecosystems, particularly in marine environments and for understudied taxa. In these contexts, reliable genus- and family-level assignments represent a pragmatic alternative that improves the interpretability and consistency of taxonomic assignments in comparative eDNA analyses. The approach presented here is therefore not intended to replace species-level assignment methods when they are applicable, but rather to complement existing pipelines by providing robust higher-rank assignments in situations where reference coverage is limited.

The results highlight the potential of learning-based approaches to address one of the major bottlenecks of eDNA metabarcoding. As reference databases continue to expand, the combined use of reference-based and learning-based methods may contribute to more reliable and reproducible taxonomic assignment across a wide range of ecological contexts.

## Figures and Tables

**Table 1:** Example of sequence transformation for our filled and aligned datasets.

(A) Raw sequence	5′-ccctgtcaaaatgcaataaaattacttaaaaccaatgcaccgacaaggggaggcaagtcgtaa-3′
(B) Filled sequence	5′-ccctgtcaaaatgcaataaaattacttaaaaccaatgcaccgacaaggggaggcaagtcgtaannnnnnnnnnnnnnnnnnnn-3′
(C) Aligned sequence	5′-nnnnnnnccctgnnntncaaaatngcaataaaattacttanaannnaccaatnngcaccgacaaggnggaggcaagtcngtaa-3′

**Table 2:** Example of a sequence transformation into 5 binary vectors.

Raw Sequence	A Binary Vector	C Binary Vector	T Binary Vector	G Binary Vector	N Binary Vector
AACGGN	110000	001000	000000	000110	000001

**Table 3:** Sequence assignment accuracy (%) obtained with our CNN on filled data and aligned data. Best results are shown in bold.

	Filled Data		Aligned Data	
	Average Accuracy	Stdev	Average Accuracy	Stdev
Family	82.76	1.83	**86.51**	1.74
Genus	94.24	2.11	**94.72**	2.56

**Table 4:** Accuracy of the CNN assignment based on varying thresholds for the number of sequences used in model training. Best results are shown in bold.

Minimum Number of Sequences Per Class	5	10	15	20
Family Accuracy	79.90	82.98	85.54	**86.51**
Genus Accuracy	76.50	88.38	94.29	**94.72**

## Data Availability

All DNA sequence data used in this study are publicly available via GenBank. The neural network architecture and training procedures are described in detail in the Methods section. Code and scripts can be made available upon reasonable request.

## Supplementary Materials

The following supporting information can be downloaded at: https://www.mdpi.com/article/10.3390/biology15030285/s1, Table S1: Family dataset. Table S2: Genus dataset. Table S3: Assignment accuracy and standard deviation on our datasets, aligned_genus and aligned_family. Figure S1: Accuracy difference between our proposal and Obitools for families. Positive values show a better accuracy on the CNN, while negative values show a better accuracy on other methods. The color gradient shows the number of sequences (testing + training) of each family. Figure S2: Accuracy difference between our proposal and Lolo for families. Positive values show a better accuracy on the CNN, while negative values show a better accuracy on other methods. The color gradient shows the number of sequences (testing + training) of each family. Figure S3: Accuracy difference between our proposal and Kraken2 for families. Positive values show a better accuracy on the CNN, while negative values show a better accuracy on other methods. The color gradient shows the number of sequences (testing + training) of each family. Figure S4: Accuracy difference between our proposal and Lolo for genera. Positive values show a better accuracy on the CNN, while negative values show a better accuracy on other methods. The color gradient shows the number of sequences (testing + training) of each genus. Figure S5: Accuracy difference between our proposal and Obitools for genera. Positive values show a better accuracy on the CNN, while negative values show a better accuracy on other methods. The color gradient shows the number of sequences (testing + training) of each genus. Figure S6: Accuracy difference between our proposal and Kraken2 for genera. Positive values show a better accuracy on the CNN, while negative values show a better accuracy on other methods. The color gradient shows the number of sequences (testing + training) of each genus.
