# Supplementary material for: Fine-Grained Assignment of Unknown Marine eDNA Sequences Using Neural Networks
Source: Biology (Basel). 2026 Feb 5;15(3):285. doi: 10.3390/biology15030285 (PMC12897059; doi:10.3390/biology15030285)
Supplement: Supplementary file 1 [file biology-15-00285-s001.zip › biology-4048518-supplementary.pdf]

Supplementary Results

Supp. Table S1 : Family dataset

FAMILY	Number of distinct sequences
Acheilognathidae	49
Anguillidae	24
Aphaniidae	26
Apogonidae	128
Bagridae	39
Botiidae	23
Carangidae	31
Carcharhinidae	70
Cichlidae	32
Clupeidae	82
Cobitidae	54
Congridae	22
Cottidae	60
Cyprinidae	215
Danionidae	44
Dasyatidae	36
Distichodontidae	29
Doradidae	37
Eleotridae	65
Engraulidae	35
Etmopteridae	47
Gasterosteidae	24
Gobiidae	425
Gobionidae	79
Labridae	169
Lacertidae	124
Leuciscidae	159
Loricariidae	226
Lutjanidae	29
Mugilidae	47
Myctophidae	32
Myliobatidae	28
Nemacheilidae	66
Nothobranchiidae	40
Odontobutidae	23
Ophichthidae	21
Percidae	42

Pimelodidae	63
Poeciliidae	60
Polypteridae	27
Pomacanthidae	31
Pomacentridae	105
Rajidae	35
Rivulidae	78
Salmonidae	21
Sciaenidae	36
Scombridae	27
Serranidae	61
Tetraodontidae	51
Xenocyprididae	37

Supp. Table S2 : Genus dataset

**GENUS**	**Number of distinct sequences**
Acheilognathidae acheilognathus	28
Anguillidae anguilla	24
Aphaniidae aphanius	26
Apogonidae apogon	24
Apogonidae ostorhinchus	30
Carcharhinidae carcharhinus	42
Etmopteridae etmopterus	39
Gobiidae chaenogobius	51
Gobiidae sicyopterus	33
Labridae halichoeres	45
Lacertidae takydromus	39
Loricariidae rineloricaria	53
Lutjanidae lutjanus	23
Nemacheilidae triplophysa	23
Percidae etheostoma	31
Polypteridae polypterus	24

Serranidae epinephelus	28

Supp. Table S3 : Assignment accuracy and standard deviation on our datasets, aligned_genus and aligned_family

	CNN		OBI		Lolo		KRAKEN	
Accuracy family	86.50	±1.74	73.25	±1.58	65.57	±1.59	44.18	±1.78
Accuracy genre	94.71	±2.56	27.28	±3.42	19.35	±2.94	16.09	±2.89

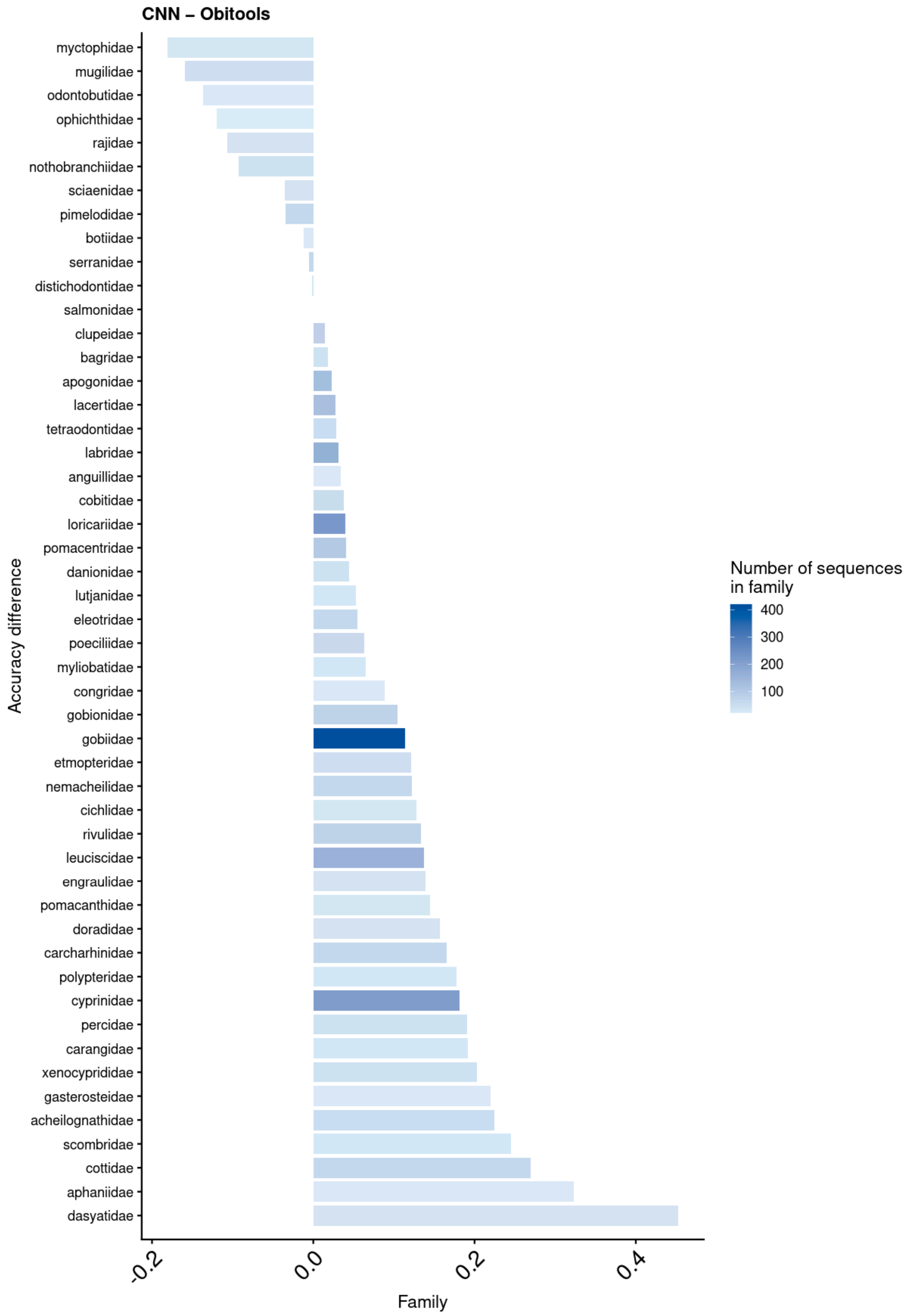

Supp. Fig. S1 : Accuracy difference between our proposal and Obitools for families. Positive values show a better accuracy on the CNN, while negative values show a better accuracy on other methods. The color gradient shows the number of sequences (testing+training) of each family.

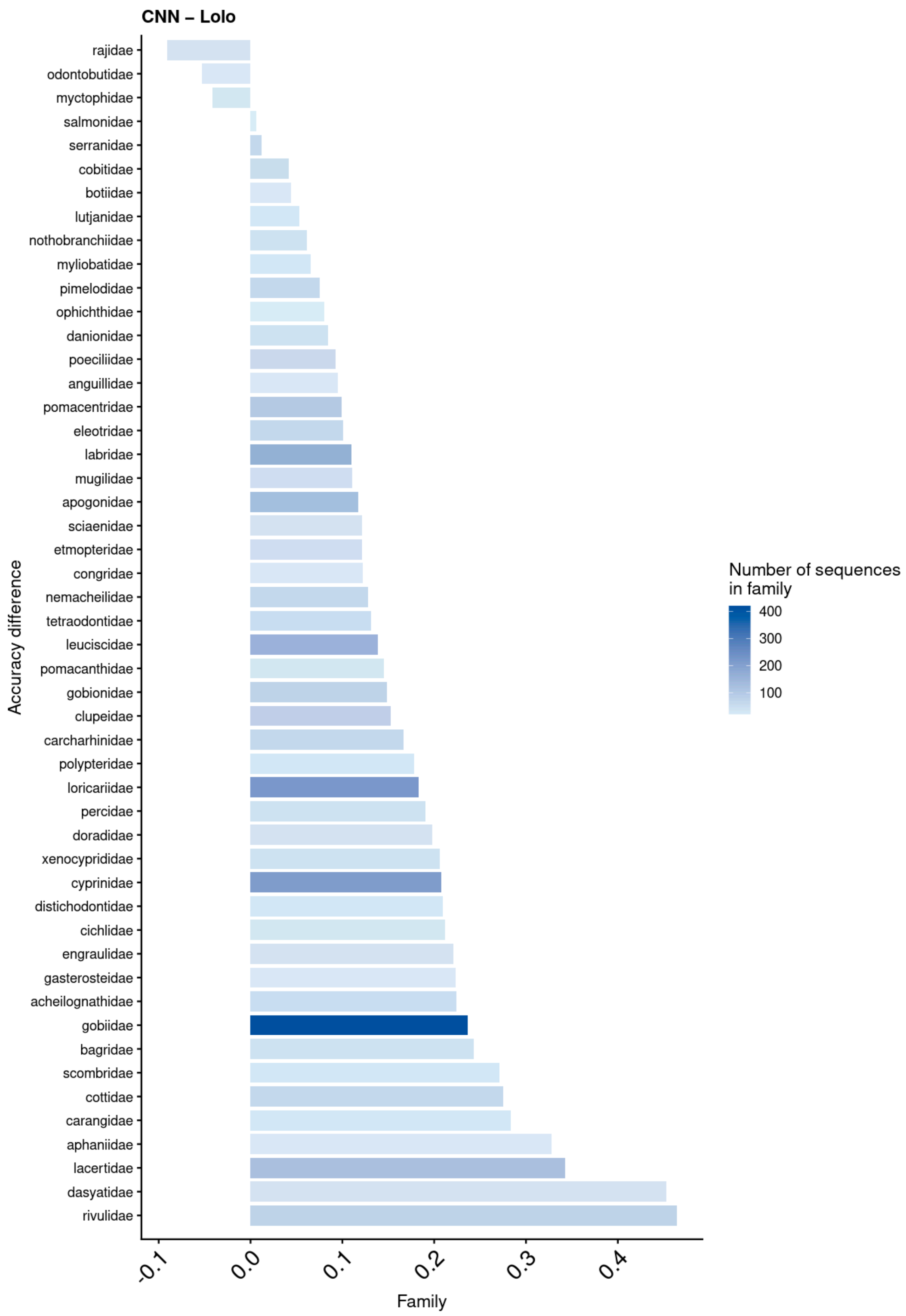

Supp. Fig. S2 : Accuracy difference between our proposal and Lolo for families. Positive values show a better accuracy on the CNN, while negative values show a better accuracy on other methods. The color gradient shows the number of sequences (testing+training) of each family.

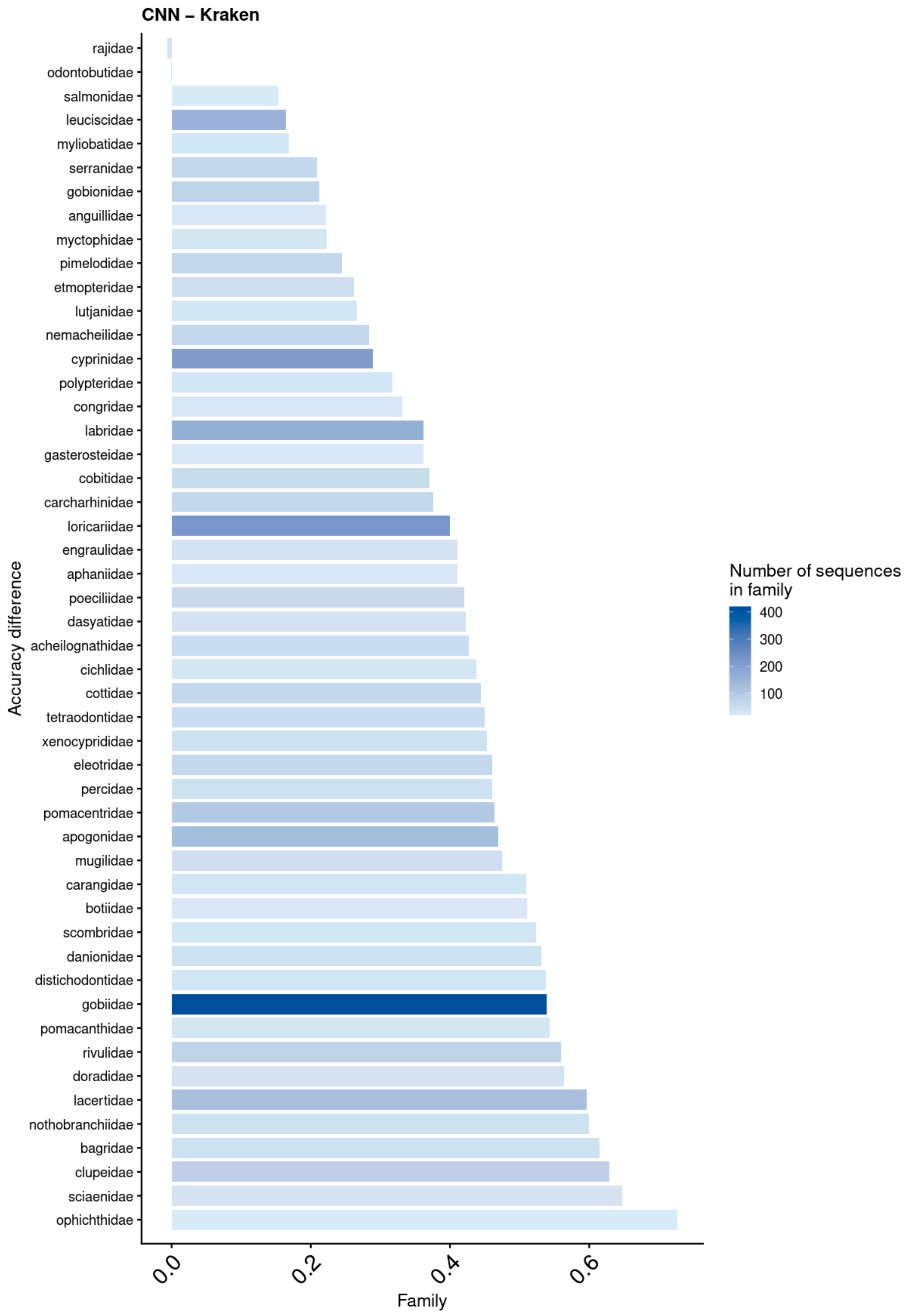

Supp. Fig. S3 : Accuracy difference between our proposal and Kraken2 for families. Positive values show a better accuracy on the CNN, while negative values show a better accuracy on other methods. The color gradient shows the number of sequences (testing+training) of each family.

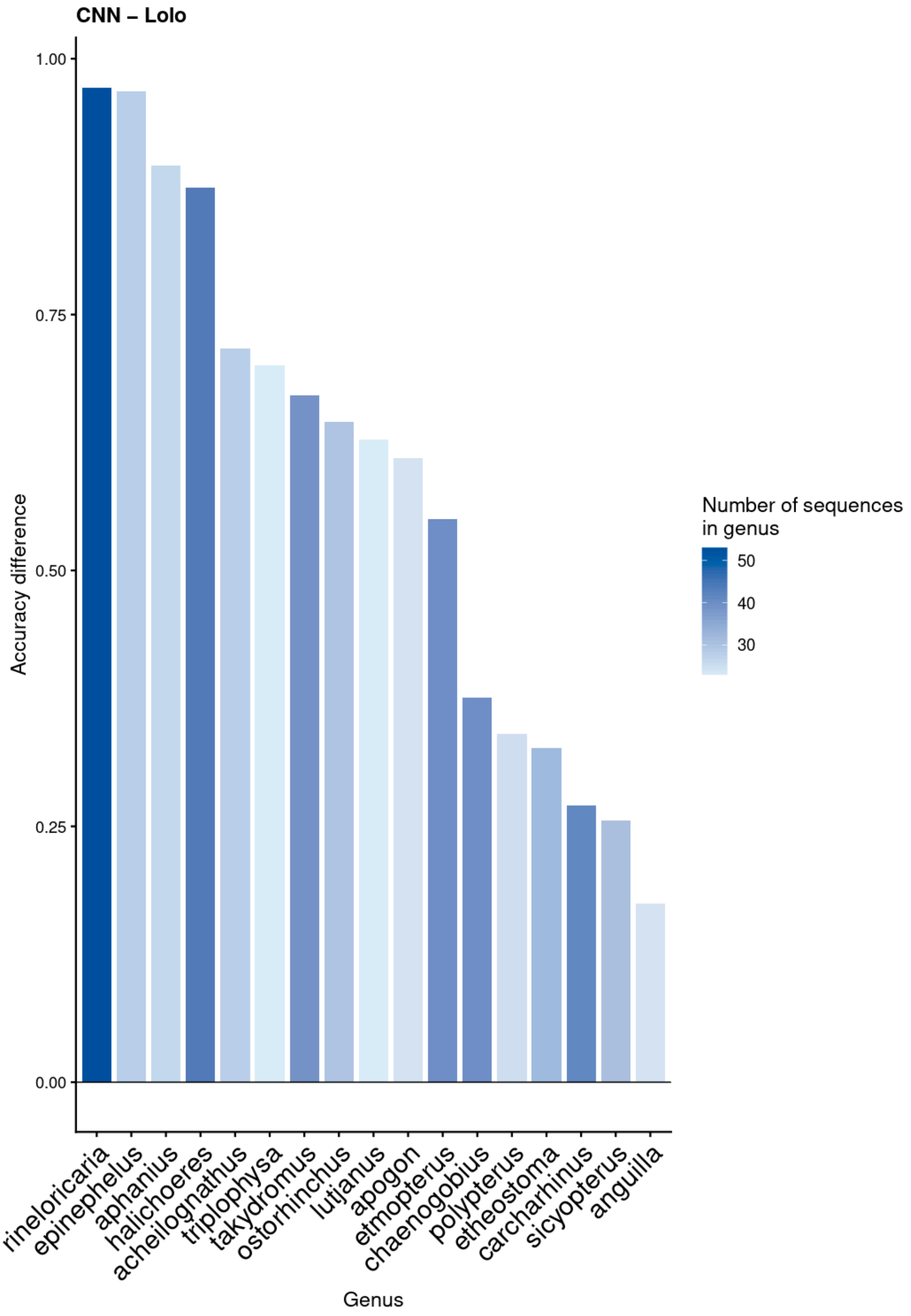

Supp. Fig. S4 : Accuracy difference between our proposal and Lolo for genera. Positive values show a better accuracy on the CNN, while negative values show a better accuracy on other methods. The color gradient shows the number of sequences (testing+training) of each genus.

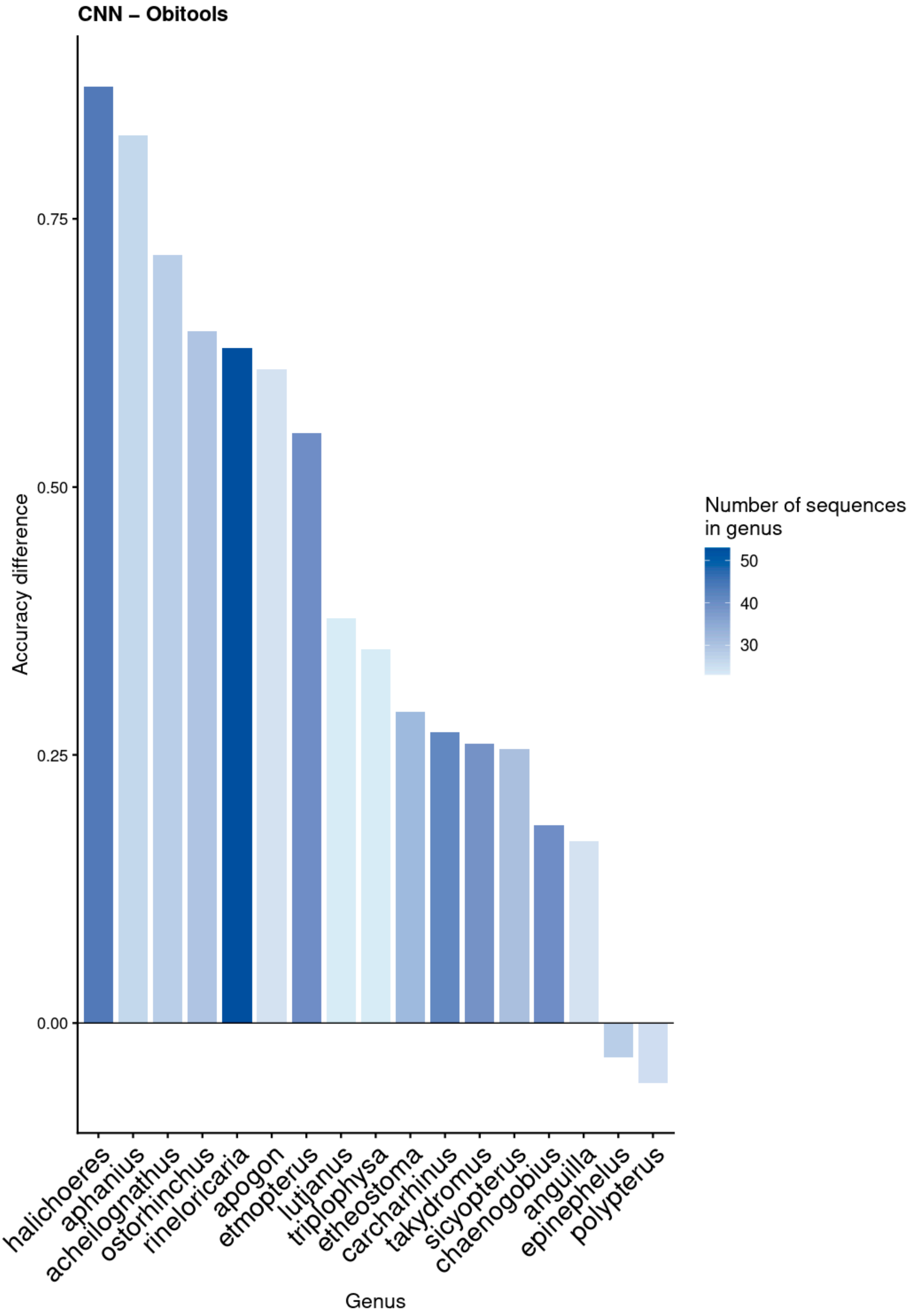

Supp. Fig. S5 : Accuracy difference between our proposal and Obitools for genera. Positive values show a better accuracy on the CNN, while negative values show a better accuracy on other methods. The color gradient shows the number of sequences (testing+training) of each genus.

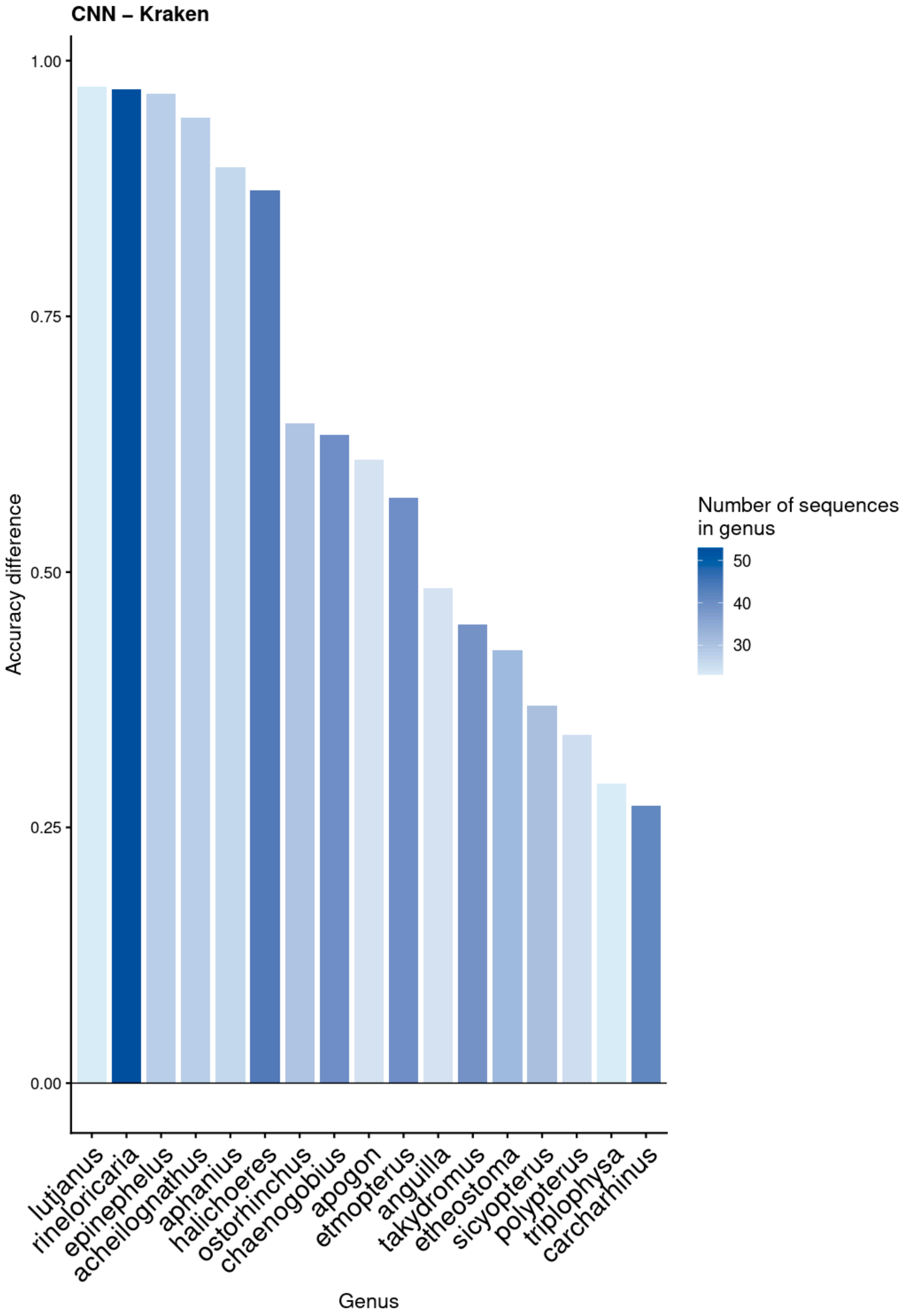

Supp. Fig. S6 : Accuracy difference between our proposal and Kraken2 for genera. Positive values show a better accuracy on the CNN, while negative values show a better accuracy on other methods. The color gradient shows the number of sequences (testing+training) of each genus.
